# An overview of FSH-FSHR biology and explaining the existing conundrums

**DOI:** 10.1186/s13048-021-00880-3

**Published:** 2021-10-30

**Authors:** Deepa Bhartiya, Hiren Patel

**Affiliations:** 1 Stem Cell Biology Department, ICMR- National Institute for Research in Reproductive Health, Jehangir Merwanji Street, Maharashtra 400012 Mumbai, India; 2 Department of Ophthalmology and Visual Sciences, University of Nebraska Medical Center, Omaha, Nebraska USA

**Keywords:** FSH, FSHR, Isoforms, Mutations, SNPs, Ovary, Testis, Uterus, Granulosa cells, Sertoli cells, Stem cells, Very small embryonic-like stem cells (VSELs), Cancer

## Abstract

FSH was first identified in 1930 and is central to mammalian reproduction. It is indeed intriguing that despite being researched upon for about 90 years, there is still so much more to learn about FSH-FSHR biology. The purpose of this review is to provide an overview of current understanding of FSH-FSHR biology, to review published data on biological and clinical relevance of reported mutations, polymorphisms and alternately spliced isoforms of FSHR. Tissue-resident stem/progenitor cells in multiple adult tissues including ovaries, testes and uterus express FSHR and this observation results in a paradigm shift in the field. The results suggest a direct action of FSH on the stem cells in addition to their well-studied action on Granulosa and Sertoli cells in the ovaries and testes respectively. Present review further addresses various concerns raised in recent times by the scientific community regarding extragonadal expression of FSHR, especially in cancers affecting multiple organs. Similar population of primitive and pluripotent tissue-resident stem cells expressing FSHR exist in multiple adult tissues including bone marrow and reproductive tissues and help maintain homeostasis throughout life. Any dysfunction of these stem cells results in various pathologies and they also most likely get transformed into cancer stem cells and initiate cancer. This explains why multiple solid as well as liquid tumors express OCT-4 and FSHR. More research efforts need to be focused on alternately spliced FSHR isoforms.

## An introduction to FSH

Follicle stimulating hormone (FSH) is a gonadotropin hormone secreted by anterior pituitary gonadotropic cells, is central to reproduction in mammals and its synthesis is regulated by gonadotropin-releasing hormone (GnRH) pulse. Activin enhances biosynthesis and secretion whereas inhibin downregulates FSH synthesis. Regulation of FSH synthesis and secretion was recently reviewed [1, 2]. It regulates the development, growth, pubertal maturation, and reproductive processes of the body. Coss [3] discussed the recent advances and current understanding of various facets of FSH biology. We also suggest readers to refer to a special issue published in Frontiers in Endocrinology in 2019 which is a compilation of 22 chapters on different aspects of FSH-FSHR biology, provides an interesting read and update of the field [4]. The present review covers this content briefly and is more focused on firstly to review the clinical relevance of FSHR isoforms and secondly it provides an explanation for the existing conundrums in the field.

## FSH structure

**Fig. 1:**
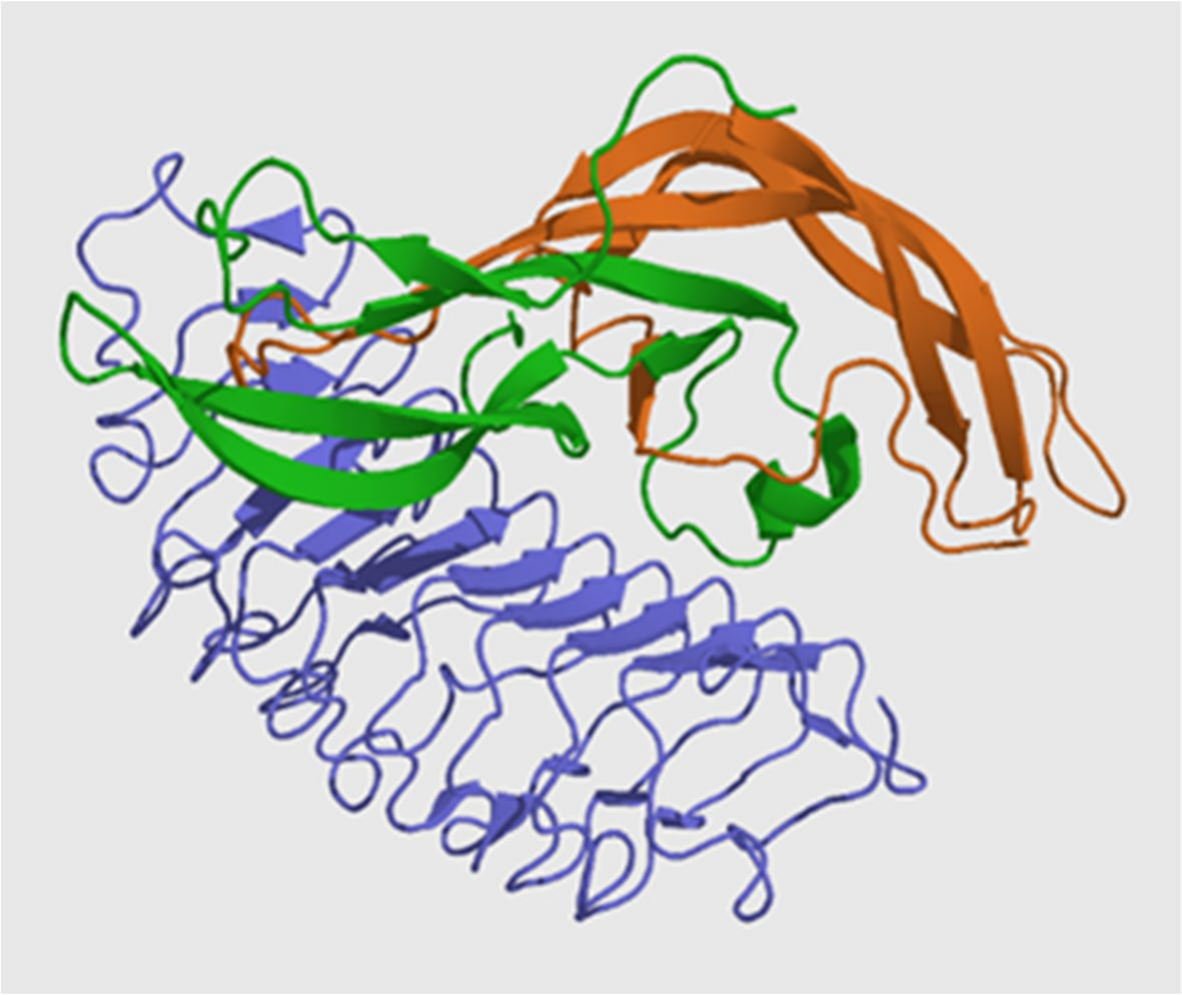
Follicle stimulating hormone: αFSH (green), βFSH (orange) with receptor (FSHR, blue). Source:  https://en.wikipedia.org/wiki/Follicle-stimulating_hormone

Structurally, FSH is a 35.5 kDa dimeric glycoprotein composed of two polypeptide units including alpha and beta subunits (Fig 1). Its structure is shared with other hormones including luteinizing hormone (LH), thyroid-stimulating hormone (TSH) and human chorionic gonadotropin (hCG). The alpha subunits of the glycoproteins LH, FSH, TSH, and hCG are identical and consist of 96 amino acids, while the beta subunits vary and are hormone specific. Both subunits are required for biological activity. The beta subunit of FSH is of 111 amino acids (FSH β), which confers its specific biologic action, and is responsible for interaction with the follicle-stimulating hormone receptor.

## Functions of FSH

 FSH classically stimulates follicular growth during development and granulosa cells and follicle growth in females and Sertoli cells function in males during adult life.

### Role of FSH in males

It is textbook information that in males, FSH stimulates Sertoli cells proliferation during development and exerts an indirect effect via Sertoli cells on the process of spermatogenesis during adult life. Sertoli cells regulate spermatogenesis by interacting with Testosterone (T) secreted by the Leydig cells. FSH stimulates the production of Androgen Binding Protein (ABP) and inhibin by the Sertoli cells that binds to T secreted by Leydig cells under the influence of LH. ABP bound T is transported into the seminiferous tubular lumen to maintain spermatogenesis. LH is considered to have a more crucial role during spermatogenesis (by resulting in the production of intra-testicular testosterone) compared to FSH. As a result, the role of FSH during spermatogenesis remains contentious. However, Huhtaniemi’s group has reported that spermatogenesis can be maintained in complete absence of LH and T by FSH alone [5–7]. Spermatogenesis remains unaffected in transgenic mice expressing activating FSHR mutation in Sertoli cells (that led to more than 10 folds of cAMP, *Fshr*-CAM; *D580H*) with minimal T production even when T was further blocked by treating with flutamide (a strong anti-androgen). These results question two paradigms of reproductive biology including role of high intra-testicular T and FSH during spermatogenesis. Apparently, spermatogenesis is possible without T in the presence of FSH alone.

### Role of FSH in females

FSH concentration shows two distinct peaks during female reproductive cycle [3]. In the estrus cycles of rodents, a surge of GnRH during afternoon of proestrus triggers a surge of both LH and FSH resulting in ovulation of mature follicles in response to LH. Later, during morning of estrus, a secondary FSH increase occurs without any change of LH. This is essential for follicles development for subsequent cycle in rodents. In humans, FSH levels increase in late luteal phase – through mid-follicular phase of menstrual cycle in addition to preovulatory rise- corresponding to recruitment of cohort of follicles to the growing pool. FSH plays a key role during ovarian folliculogenesis and antral follicle development and, in combination with luteinizing hormone (LH), stimulates preovulatory follicular growth. In contrast, the primordial follicles are thought to be FSH-independent. Primordial follicles develop up to the late preantral stage in the ovaries of mice lacking functional gonadotrophin releasing hormone (GnRH) [8, 9], FSH beta-subunit [10] or FSH receptor [11, 12]. FSH affects granulosa cells proliferation which synthesize estrogen crucial for follicle maturation, growth and maturation of antral follicles and prepares the dominant follicle for ovulation in response to LH surge.

Normally in humans only one follicle becomes dominant and survives to grow to 18–30 mm in size and ovulate, the remaining follicles in the cohort undergo atresia. The increase in serum estradiol levels cause a decrease in FSH production and LH surge. The decrease in serum FSH level causes the smaller follicles in the cohort to undergo atresia as they lack sufficient sensitivity to FSH to survive. At the end of the luteal phase, there is a slight rise in FSH that seems to be of importance to start the next ovulatory cycle. Deficiency of FSH in females results in absent or incomplete pubertal development and blocked folliculogenesis prior to antral stage and infertility during adult life. In perimenopausal women, number of small antral follicles recruited in each cycle decrease in numbers and as a result inhibin B is not able to fully lower FSH and the serum level of FSH begins to rise. Eventually, the FSH level becomes so high that down-regulation of FSH receptors occurs and by post-menopause, remaining small secondary follicles no longer express FSH or LH receptors.

It is still debated whether early primordial follicular growth is FSH dependent or not. Moreover, with the presence of stem cells in adult ovaries, the ovarian biology becomes more complex and a role for FSH during neo-oogenesis and primordial follicle assembly from the stem cells is yet to be deciphered convincingly. McGee et al. [13] showed that FSH responsive follicles may exist earlier to antral follicles. FSH not only advances development of antral follicles but also affects smaller growing follicles several cycles prior to becoming the leading cohort. Roy and Albee [14] studied perinatal FSH role in primordial follicle formation which appear on D7-8 after birth in hamster ovaries. Injecting FSH specific antibodies in pregnant hamsters resulted in significant reduction in PF numbers (2.4% vs 25%) which could be reversed by injecting eCG (18%). Results provided first direct evidence that FSH action during fetal ovarian development is critical for primordial follicle formation. Allan et al. [15] showed that FSH affects follicle reserve as well as their recruitment. Treating adult, hypogonadal, gonadotropin deficient mice (hpg) expressing transgenic human FSH showed four folds increased ovary weight and significantly elevated total primordial follicle numbers in age matched ovaries in addition to more numbers of secondary and antral follicles [numbers of primordial follicles: wild type ovaries (2043 + 195), hpg mice (2079 + 391), and Tg-FSH hpg (4209 + 457)]. Our group has reported FSHR on endogenous, tissue-resident stem cells in adult ovary and testes and FSH stimulates the stem cells to undergo proliferation, asymmetrical, symmetrical and clonal expansion [16–20].

## FSH treatment for infertility

Different types of gonadotropin formulations are available to stimulate the ovaries [21]. Initially gonadotropin (hCG) was extracted from the pituitaries of animals and human cadavers and later from the urine. Now several recombinant and biosimilar FSH are available for clinical use and were recently reviewed [22].

### Male infertility

Hypogonadotropic hypogonadism in men with insufficient secretion of FSH and LH by pituitary gland, are subjected to treatment with FSH (150–225 IU FSH 2–3 times a week along with 1000- 2500 IU of hCG twice a week) with high clinical efficiency [23, 24]. FSH is now also being suggested to be used to treat normo-gonadotrophic men with idiopathic impairment of spermatogenesis and has shown beneficial results in almost 50% of cases [22, 23]. High FSH dose (300 IU per day) stimulates Sertoli cells and provide a stronger support to germ cells proliferation and maturation [25] and use of 150 IU on alternate days leads to significant increase in sperm numbers and improvement of sperm morphology in Italy [26]. A meta-analysis of the published literature suggests that FSH treatment to male partners of infertile couples, both spontaneously and during assisted reproduction, improves pregnancy rates [24, 27]. However, this treatment is not yet offered as a method of standard care to men with idiopathic infertility.

### Female infertility

Women in infertility clinics are routinely treated with FSH to stimulate the ovaries to collect multiple eggs for assisted reproduction. Human menopausal gonadotropin (HMG) and recombinant FSH are routinely used in controlled ovarian stimulation in infertility treatment. By increasing the blood levels of FSH, several follicles grow at approximately the same rate allowing collection of more than one mature egg. As per current understanding, new eggs are not created but rather, eggs are rescued by high FSH, that would otherwise undergo atresia.

## Disorders of FSH action

Both inactivating and activating mutations have been reported in FSHβ. Activating mutations are associated with normal spermatogenesis in men and spontaneous OHSS in women.

### Males

Inactivating mutations in men lead to azoospermia while spermatogenesis was not affected in the knockout mice. FSHβ-/- mice have normal development, are fertile, testes are small in size with 75% reduction of sperm. Only 3 men have been described with mutations in FSHβ that resulted in complete loss of immuno- and bioactivity and all were azoospermic. First man with mutation was reported in 1998 had normal puberty, small testes with varying degree of spermatogenic failure [28]. T levels were low, LH was mildly elevated and FSH was undetectable. Phillip et al. [29] reported the second FSH-deficient man with no evidence of pubertal development, small testes, low T, elevated LH and low FSH. The third male with FSHB mutation was characterized by Layman et al. [30]. He had normal puberty, infertility, and azoospermia.

### Females

Follicular development up to the primary and early antral stages was observed in the mice ovaries in the absence of pituitary FSH. Fshb-/- mice have low FSH, lack estrus cycles and are sterile. Fshb ± heterozygote mice have normal estrus cycles and are fertile. Five different mutations in FSHβ have been reported in 6 women. Four of the mutations studied in vitro resulted in unmeasurable immune- and bioactive FSH. First patient with FSHB mutation was described by Matthews et al. [31]. She was a 27-year-old female with primary amenorrhea, low serum FSH, and high LH. At the age of 13, she had no breast development but was subsequently treated with estrogen. She later conceived with exogenous gonadotropin therapy. Second female presented with absent breast development, primary amenorrhea, low FSH, undetectable estradiol, elevated LH levels and no pituitary tumor. 4 more females with FSHB mutations were reported with partial breast development. All the 6 females shared certain features including primary amenorrhea, low serum FSH, low serum estradiol, and elevated LH.

## FSH mediates action via FSH receptors (FSHR)

**Fig. 2:**
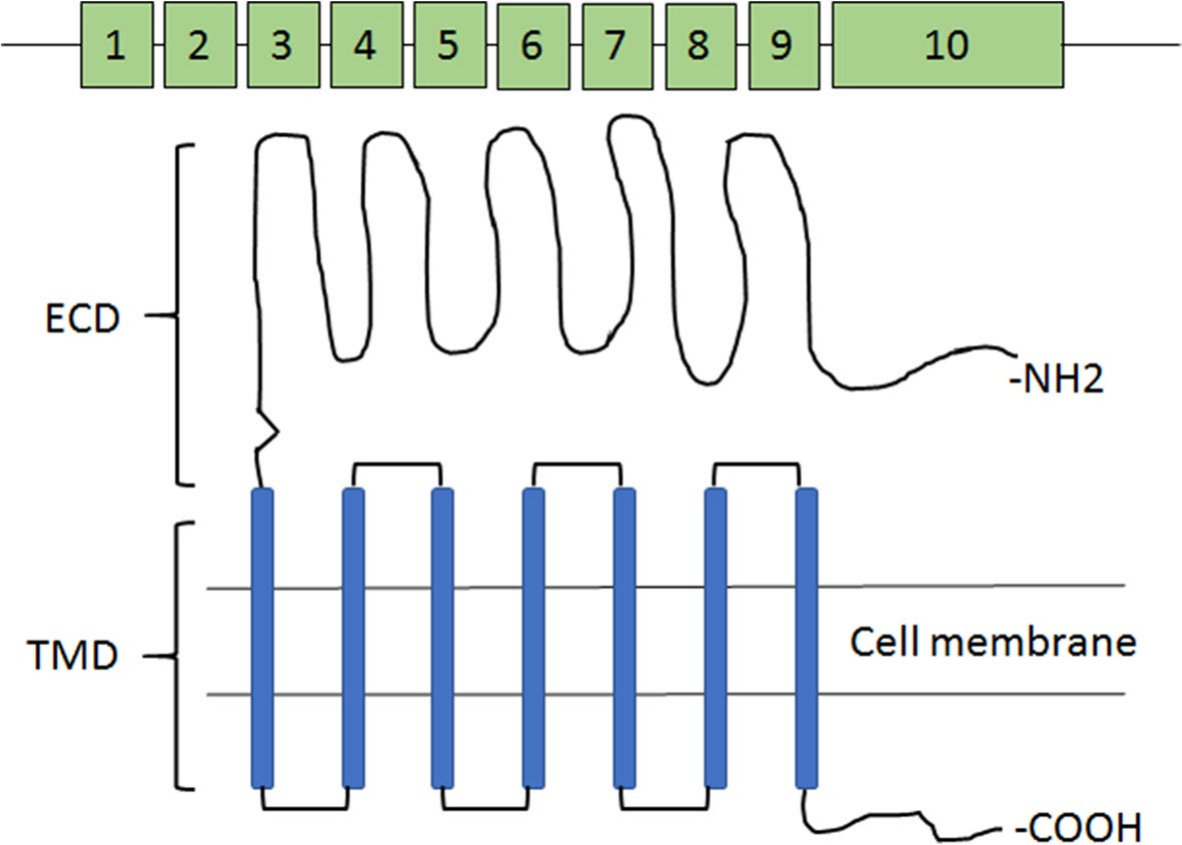
Schematic representation of FSHR gene and protein

Human FSHR is a G protein coupled receptor with a long ECD, a 7 transmembrane domain, 3 short intracellular loops, 3 extra loops and an intracellular tail (Fig. 2). FSHR binds to FSH by the very large ECD. Its molecular weight is approximately 75 kDa. The FSHR protein comprises 695 amino acids, including a 17 amino acid signal peptide sequence; the mature receptor consists of 678 amino acids with a predicted molecular weight of approximately 75 kDa and three to four potential glycosylation sites [32]. The human FSHR gene is located on chromosome 2p21-p16 and is a single copy gene of 54 kb in length. The human FSHR gene contains 10 exons and 9 introns and a promoter region. The extracellular domain is encoded by nine exons; the C-terminus, transmembrane domain, and intracellular domain of the extracellular domain are all encoded by exon 10. Multiple isoforms of FSHR have been reported and FSHR has been also expressed on extragonadal tissues including placenta, uterus, prostate, bone tissue and ovarian epithelium as well as ovarian cancer. FSHR has also been found to be selectively expressed on the surface of many tumor blood vessels, and related to tumor metastasis.

## Mutations, Polymorphisms and Isoforms of FSHR

Defects in FSHR structure as a result of mutations, polymorphisms and other anomalies result in various disease conditions.

Mutations: Both activating and inactivating mutations have been described in both the sexes and cause alteration of reproductive function, even though the phenotype is often more severe for female fertility. Activating mutations confer to FSHR a higher responsiveness to FSH, making it constitutively active even in the absence of the ligand. Inactivating mutations reduce the receptor function up to a total block, altering either the formation of the receptor-ligand complex, or FSH signal transduction. FSHR inactivating mutations cause primary or secondary amenorrhea, infertility, and premature ovarian failure (POF), whereas activating mutations can predispose to ovarian hyperstimulation syndrome (OHSS).

Polymorphisms: Around 1800 SNPs of the FSHR gene have been reported in the SNP database (http://www.ncbi.nlm.nih.gov). SNPs are located either in the coding regions (exons, 8 SNPs) or within intronic regions of exons. Only 1 SNP is located in the 5′ untranslated region of the FSHR mRNA position − 29 (ss2189241). Of the eight SNPs within coding regions, 7 are located in exon 10 at codon positions 307, 329, 449, 524, 567, 665, and 680. Six of the latter SNPs eventually result in amino acid substitution and are therefore non-synonymous. The two best characterized polymorphisms as far as their allele frequencies and ethnic distribution is concerned are the Ala307Thr (rs6165) and the Ser680Asn (rs6166). These SNPs influence FSHR protein responsiveness to exogenous FSH, and finally affect the effectiveness of in vitro fertilization (IVF) treatment as well as the likelihood of developing a severe OHSS as a consequence of superovulation. SNPs at 307 and 680 of exon 10 are the most common in FSHR (Fig. 2) [33]. Banerjee et al. [32] have compiled various pathologies due to FSHR dysfunction in men and women and evidently these are more severe in women resulting in infertility compared to men where they lead to subfertility.

**Fig. 3:**
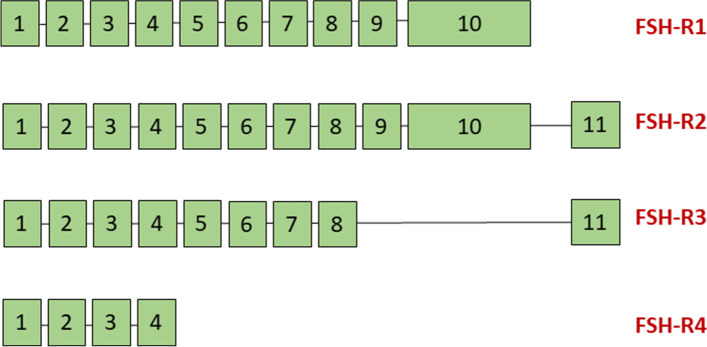
Four alternately spliced FSHR isoforms as described earlier [35]

FSHR isoforms: FSH is pleiotropic in nature and is responsible for multiple diverse functions including cellular growth, differentiation and steroidogenesis resulting in the formation of single large follicle or daily production of millions of sperm. In mammals large numbers of functional proteins are encoded by only 23 to 25,000 genes possibly via split genes and alternative mRNA splicing which leads to the creation of multiple proteins from a single gene. Nearly half the genes undergo alternate splicing [34]. Four different FSHR isomers exist (Fig. 3) due to different splicing mechanisms of FSHR exons [35, 36]. Zhou et al. [37] reported 2 additional FSHR isoforms in the human ovary surface epithelial cells. All the reported functions cannot be performed through a single receptor. Thus, the pre-mRNA of a single large gene coding for FSHR undergoes alternate splicing creating 4 different isoforms with similar N-terminus and different c-terminus. They have been reported in mice, rats, sheep, bovine species as well as in humans. Human ovarian granulosa cells show the presence of all 4 transcripts. However, little evidence at present that these receptor isoforms have any clinical relevance in humans.

### FSHR1

The FSHR1 variant is the full-length form of FSHR. It is the canonical G protein coupled receptor. The structure of FSHR1 typically includes a large extracellular N-terminal domain that binds to FSH and an intracellular C-terminal domain segment, 7 α-helical membranes are alternately connected by extracellular and intracellular circulation. FSHR1 is expressed only in ovarian granulosa and testicular Sertoli cells and its primary function is to participate in follicular development and granulosa cell differentiation. R1 is the full-length FSHR comprised of 10 exons.

### FSHR2

Unlike FSHR1, FSHR2 retains the extracellular and transmembrane domains of the receptor, but not the intracellular domain. This variation retains the high affinity binding of FSHR to FSH, but lacks the ability to activate G protein when bound to FSH. R2 has the 10 exons but has a different C-terminus. No biological function is reported so far.

### FSHR3

FSHR3 subtype has a single transmembrane domain, and the overall topology of FSHR3 is consistent with that of the growth factor I receptor. FSHR3 promotes growth by activating MAPK and Ca -dependent channels. FSHR3 may play an important role in promoting mitotic activity and cell growth. R3 has exons 1–8, but is truncated, and appears to function as a cytokine/growth factor type receptor. Has lots of biological functions which are discussed ahead.

### FSHR4

FSHR4 lacks any transmembrane domain and is called soluble FSHR. FSHR4 stabilizes or blocks the binding of hormones to FSHR by binding to FSH in the extracellular matrix. FSHR4 may function like the insulin-like growth factor (IGF-1) binding protein. But its exact function is unclear. R4 has only exons 1–4 and is a truncated receptor with uncertain function.

## Clinical relevance of FSH/FSHR mutations and polymorphisms on reproductive functions and pathologies

Research has been undertaken over last two decades to study a possible role of mutations/single nucleotide polymorphisms of FSHR in modulating reproductive functions and also for providing a genetic basis for various pathologies in both men and women. However, the data remains controversial and the clinical relevance of FSHR mutations/ polymorphisms remains limited. Larger studies have failed to confirm any association between the FSHR genotype and serum FSH [38]. Molecular mechanism by which FSHR SNPs could lead to a phenotype also remains poorly understood. Combination of FSHR and FSHβ genotypes together seems to have a stronger impact but there is not enough evidence to provide practical clinical recommendation even after combining FSHR and FSHβ genotypes [39 40]. Whatever little correlation, also falls flat with increased maternal age. Systematic reviews fail to arrive at any consensus on the ideal dose of FSH to stimulate ovaries for a favorable end point. Besides, SNPs show variations specific to ethnicity. Thus, their relevance for reproductive functions and to provide genetic basis for various pathologies remains questionable. No clear clinical benefit of screening patients for FSHR SNPs have yet emerged and the results are not consistent.

- Simoni’s group [41] for the first time reported that FSHR haplotype with 2 SNPs in exon 10 could determine serum FSH levels. They showed that women with Ala307-Ser 680 were less sensitive to FSH and required more FSH for ovarian hyperstimulation. This is the only SNP which is extensively studied and is associated with a need for higher FSH dose to achieve similar estradiol peaks as in normal women.
- Determining the dose of FSH to attain optimum response is one of the ongoing challenges in the field of infertility management in IVF clinics. It was earlier envisioned that FSHR genotype could influence the ovarian response to FSH stimulation and thus might have important therapeutic implications for fertility treatment. SNPs at positions 307 and 680 in exon 10 of the FSH receptor gene were thought to predict ovarian response to FSH stimulation. But serum FSH levels do not differ between FSHR genotypes in older women who are present in majority in ART clinics. With the increasing demand of infertility treatment, therapies using FSH are evolving rapidly- mostly on empirical basis, and protocols for controlled ovarian stimulation are adjusted based of patient needs.
- Clinical pregnancy rate in women undergoing IVF-treatment in Asn/Asn is significantly higher compared to Ser/Ser women [42]. However, another study shows opposite results, with higher pregnancy rates in women with the Ser/Ser genotype using a similar study design.
- Kerkela et al. [43] did not find any SNPs in coding region of FSHR gene predisposing women to ovarian hyperstimulation syndrome (OHSS) which is a disorder showing exaggerated ovarian response to FSH. However, no consensus and it is thought that several other factors like VEGF, interleukins etc. may also be involved and result in OHSS.
- Polycystic ovarian syndrome (PCOS) is an endocrine disorder which affects about 10% of women during reproductive age. Despite a large number of studies, genetic basis of this pathology remains unidentified. No significant correlations in the distribution of FSHR polymorphisms and FSHB genotype have been reported in women with PCOS). Ovarian response and PCOS severity may be influences by SNPs in Exon 10 of FSHR but do not reflect disposition to the disease. Similarly, association of PCOS with FSHB genotype remains questionable [44, 45].
- Premature ovarian failure (POF) or insufficiency (POI) is loss of ovarian function and the occurrence of amenorrhea before 40 years of age. Extensive genetic studies have been reported trying to identify mutations/polymorphisms in several genes including FSH/FSHR. Mutation in FSHR gene in exon 7 was reported associated with hypogonadotropic ovarian dysgenesis [46]. FSHR is implicated in POF since FSHR knockout mice with block in folliculogenesis in primary stage show classical POF phenotype with elevated FSH [47]. Also, genes involved in the negative feedback control of FSH like inhibin subunit genes have shown a functional mutation resulting in FSH increase, follicle depletion and POF [48]. But several meta-analyses have concluded that FSHR polymorphisms are not associated with POF [49].
- Endometriosis occurs due to ectopic growth of endometrium outside the uterus. It was thought to have a strong genetic component and several association studies have been reported in the literature. Homozygous FSHR polymorphism Asn680 have protective effects on development of endometriosis in Chinese women [50]. However, results need to be confirmed by others and the findings may be coincidental. There is no convincing evidence to link FSH/FSHR gene with endometriosis. In fact, after several recent GWAS studies, it has been suggested that rather than genetic factors, somatic mutations acquired during lifetime may have a greater role to explain initiation of endometriosis [51].
- FSH/FSHR role in male fertility and spermatogenesis is well studied. FSHR-mediated signaling is required for normal testicular response to FSH. Common FSHR polymorphisms in exon 10 and the promoter of FSHR have been extensively studied in male infertility [52] but there is no clear association of male infertility with FSHR SNPs so far [53]. Ahda et al. [54] reported protective role of 3 FSHR SNPs G29A, A919G, A2039G against male sterility in German men. These results were later confirmed by a meta-analysis in 2010 [55]. However, Balkan et al. [56] did not find any association of FSHR polymorphisms on spermatogenesis and FSH levels in Turkish infertile men.
- Stimulating effects of FSH on growth of ovary surface epithelium cell lines and the findings that overexpression of FSHR on CHO cells leads to proliferation suggested a possible role of FSH/FSHR in ovarian cancers  [57]. Yang et al. [58]  reported an association between FSHR isoforms with susceptibility to ovarian cancer in Asian population but similar results were not observed in Caucasian population [59].
- Testicular cancer has also been studied for a possible co-relation with FSHR polymorphisms in Italian patients. Ala307/Ser680 haplotype was found lower in control group suggesting an association with decreased risk [60]. But the results have not yet been replicated by other groups.

## Relevance of studies reported on FSHR isoforms

Nearly half the genes undergo alternate splicing [34] and thus studies on splice variants and understanding their functions in normal physiology and in pathologies is crucial. FSHR isoforms remain poorly studied and further focus on them is required especially in the backdrop of the discussion above that FSH/FSHR mutations and polymorphisms have failed to show any strong association with various reproductive pathologies. The field is still nascent with huge scope for further research. SNPs are studied on the DNA whereas isoforms are studied on the RNA isolated from cells of interest. Some of the studies highlighting an important role of FSHR isoforms are listed below.

- Babu et al. [61] treated immature 21 days old mice with PMSG (5 IU) and showed a two-fold increase in FSHR-3 expression in the ovaries over FSHR-1 after 24 or 48 h at both mRNA and protein level.
- Song et al. [62] reported FSHR isoforms in infertile men in an infertility clinic. They reported three alternately spliced variants of human FSHR but it was not clear if there was any association with spermatogenesis defects.
- Choi et al. [63] reported that treatment with FSH results in activation of the MAPK cascade and activated MAPK-phosphorylated Elk-1 in immortalized ovarian surface epithelium (IOSE) cell lines (normal), IOSE-29 (preneoplastic) and IOSE-29EC (neoplastic and tumorigenic). These results indicate that FSH exerted a growth stimulatory effect in normal, preneoplastic, and neoplastic OSE cells via MAPK pathway. The cAMP pathway which is associated with canonical FSHR-1 was not affected. FSHR-3 is a growth stimulatory isoform and acts via MAPK. Since cAMP is not involved suggests no role of canonical FSHR1 and FSHR3 is implicated during proliferation of OSE cells.
- It has been shown that FSH activates ovarian cancer cell proliferation by acting on FSHR-3 rather than the canonical FSHR-1. Li et al. [64] and Sairam et al. [65] demonstrated this using human OSE cell line ID8.
- Gerasimova et al. [66] studied ovarian response to FSH stimulation in IVF clinic by focusing on FSHR mRNA in the cumulus cells by RT-PCR. 4 spliced products were observed in 13 of 35 women. All affected extracellular ligand-binding portion of the receptor without causing a frameshift, showed decreased cAMP activation in vitro. Results suggested FSHR isoforms provide a real correlation of FSH with ovarian response in IVF clinics.
- Sullivan et al. [67] studied FSHR isoforms in ovarian follicles of untreated, normal cyclic, mature ewes. Follicles of different stages of development (small, medium, large and preovulatory) were separated, RNA extracted and subjected to qRT-PCR for FSHR isoforms. They observed that FSHR3 was the predominant isoform in all the developing follicles and LHR were maximal in pre-ovulatory follicles. Results showed that in addition to the canonical, G protein-coupled form of the FSHR, alternatively spliced variants of the FSHR may participate in follicular dynamics during follicular waves of the sheep estrous cycle.
- Zhou et al. [37] cloned alternatively spliced FSHR-2 and FSHR-3 in human OSE cells. Both the isoforms were expressed at low level, but were detected in cells from the follicular fluid of 30 women.
- Patel et al. [17, 18] reported alternately spliced FSHR3 isoform on ovarian stem cells. Similar action of FSH via FSHR3 was also noted in mouse testes [19].
- Karakaya et al. [68] showed presence of FSHR isoforms in women who were poor responders to FSH treatment. None of the splice variants could initiate cAMP signaling despite high FSH doses.
- Mamas et al. [69] reported that alternately spliced variants of FSHR may be associated with poor or high response to exogenous FSH.
- Song et al. [70] investigated FSH induced proliferation of epithelial ovarian cancer cells is not mediated via cAMP.
  *To conclude, rather than genetic changes (mutations, SNPs) in the canonical FSHR, differential expression of FSHR isoforms may have a greater relevance to explain various ovarian/testicular biology and pathologies. But much more work is required in the field.*

## Existing conundrums in our current understanding of FSH-FSHR biology and their explanations

Although FSH is central to reproduction, it is rather surprising that several aspects of FSH/FSHR biology need to be still better understood and require further research. Coss [3] published a review on the known knowns and known unknows regarding FSH-FSHR biology. FSHR expression has been reported in several extragonadal normal tissues including uteroplacental tissue (maternal decidua and placenta), placenta (maternal decidua and amnion removed), umbilical tissue, normal endometrium, myometrium, cervical glandular epithelium. Besides, FSHR are also expressed on cells in bone and fat osteoclasts. The major confounding issue is expression of FSHR in tumor samples from multiple organs [71–73]. FSHR is reported to be aberrantly expressed in the endothelial cells of tumors affecting various organs including prostate, breast, colon, pancreas, urinary bladder, kidney, lung, liver, stomach, testis, and ovary [74] and pancreatic neuroendocrine tumors [75]. Surprisingly, the surrounding non-malignant normal cells within cancer tissue are devoid of FSHR expression. Reproductive biologists have expressed concerns over extragonadal expression of FSHR and pointed out several technical shortcomings which could result in non-specific extragonadal expression of FSHR [71]. It is alleged that extragonadal FSHR expression is possibly an artefact that needs to be ignored. But we strongly believe these are genuine findings waiting for possible explanations/interpretations. Various concerns raised have been listed below along with possible explanations.

### Experiments performed only on the basis of immuno-histochemistry using poorly characterized FSHR antibodies. It is not possible to detect FSHR by Western blotting as protein is very less (~ 0.054 fmol/mg of total protein) in ovary and about 0.04 pg/μg of total protein in testes [71]

Different antibodies have been used to study FSHR immuno-expression and consistently FSHR expression has been reported on extragonadal tissues. Few groups have also confirmed FSHR by Western blotting in tumor tissues and reported multiple alternately spliced isoforms. Sardella et al. [75] showed presence of FSHR isoforms ranging from 240 to 55kD in pancreatic neuroendocrine tumors by Western Blotting. Earlier Sairam’s group has reported FSHR isoforms by Western blot [35, 61].

**Fig. 4:**
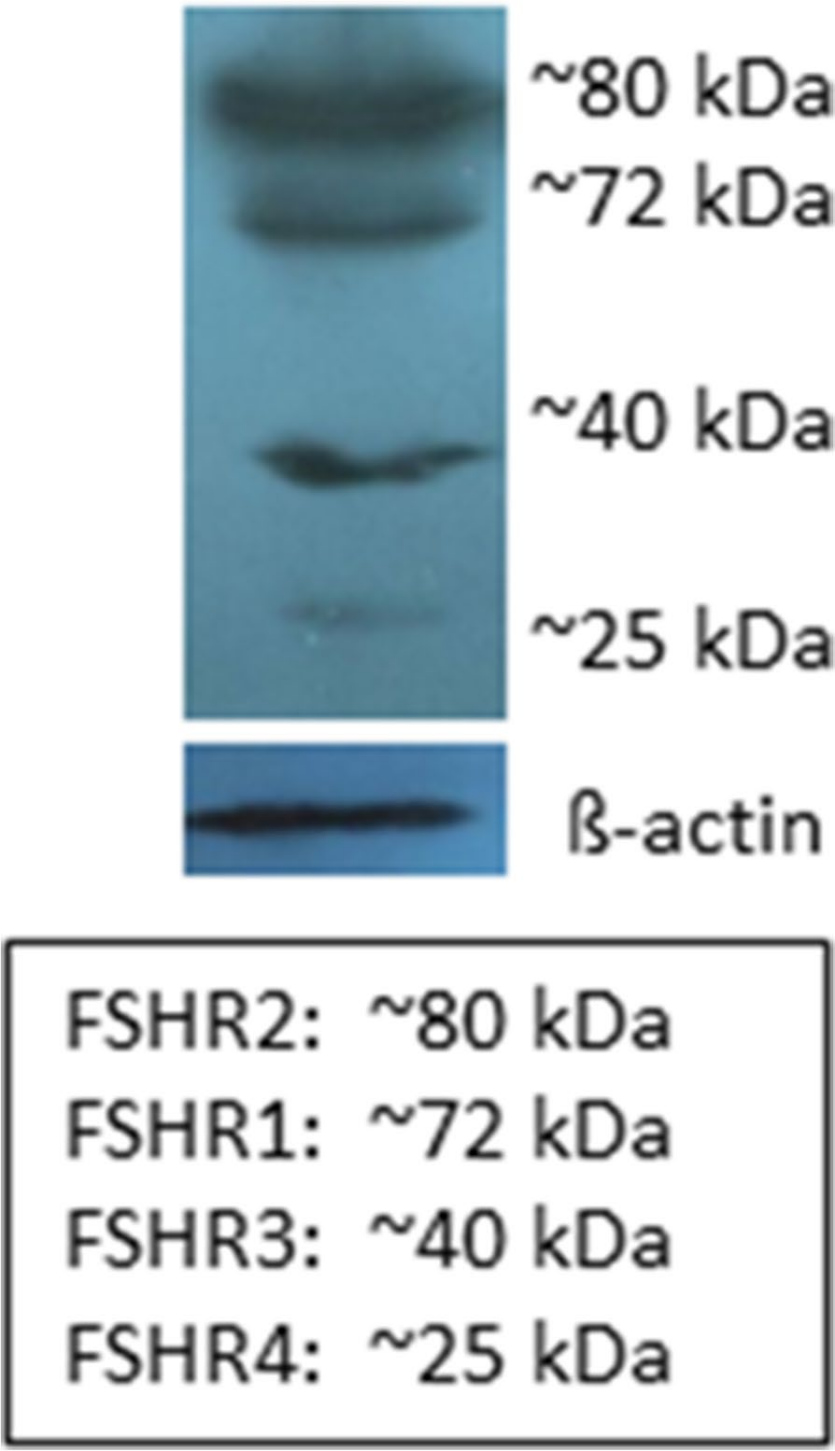
Western Blotting showing FSHR isoforms in adult mouse uterus [76]

Our group has reported 4 FSHR isoforms in adult mouse uterus [76] (Fig. 4). A polyclonal antibody raised against the N terminal of the FSHR (ABCAM) was used for the study. This antibody revealed all the 4 alternately spliced FSHR isoforms. BLAST analysis of the peptide immunogen used to raise the antibody showed no homology with other human proteins. For the Western blot experiment, protein was extracted from mouse uterus after treatment with FSH which must have increased FSHR expression. Pre-incubating FSHR antibody with specific peptide (against which it was raised) during immuno-localization studies completely abrogated FSHR expression thus proving that FSHR expression on the stem cells is specific.

Thus, it is possible to detect FSHR by Western blotting. Antibody selected should be from the N terminal which is common to all the isoforms. Using antibodies against C terminal will possibly fail to detect the isoforms. FSHR is not a unique antigen against which antibodies cannot be raised. We have also successfully used this antibody (ABCAM) to study FSHR expression on stem cells from multiple tissues including ovary [17, 18], testis [19], uterus [77], bone marrow [78]. Functional FSHR have been reported on both murine and human hematopoietic cells in vitro as well as in vivo [79, 80]. Only limitation at present is that the currently available FSHR antibodies fail to discriminate various isoforms during immuno-localization experiments.

### Discrepancy between hardly traceable or absent Fshr transcripts and strong FSHR expression. This raises doubt on non-specific IHC results or it is likely that FSHR transcripts get rapidly degraded [71]

This could be explained because the stem cells in adult tissues harbor both Fshr transcripts and the protein. FSH has a direct role in stimulating stem cells to divide [18–20]. Once stem cells initiate differentiation, FSH has no further role, Fshr are no longer required, FSHR protein is expressed and eventually gets degraded. As a result, fully differentiated adult cell types may or may not express FSHR but it will prove difficult to detect Fshr transcripts which indeed exist in the stem/progenitor cells and help in the translation of FSHR protein. RNA/protein extracted from intact tissue may not provide information at the level of stem cells as they comprise < 1% of total cells.

Another reason why it has proved difficult to detect the Fshr transcripts is related to primer design- from which region of the gene the primers are designed to detect the transcripts. As discussed above, FSHR has 4 alternately spliced isoforms (Fig. 3). Careful designing of primers is essential to detect all the Fshr transcripts. Primers specific for canonical Fshr1 will not detect transcripts specific for other transcripts. Fshr3 is pre-dominantly expressed in the stem cells rather than the canonical Fshr1. When the stem cells from ovary, testis and uterus are treated with FSH, Fshr3 is upregulated rather than the canonical Fshr1. If primers are designed against exon 10, it will never pick up increased Fshr3 expression [18, 19]. FSHR will be detected at protein level but transcripts for Fshr3 will remain undetected using primers specific for Fshr1. It becomes important to study the contribution of alternatively spliced Fshr isoforms which has been ignored till date and led to the existing confusion in FSH/FSHR biology in ovary/testis.

A careful review of literature reveals that primer selection for RT-PCR and in situ hybridization from different exons of FSHR gene and presence of alternatively spliced FSHR isoforms has led to the erroneous conclusion and text book information that initial follicle growth is gonadotropin independent. Earlier studies in late nineties in sheep [81] and humans [82] used techniques like in situ hybridization and RT-PCR to study FSHR expression on ovarian follicles of different stages of development. FSHR mRNA was observed only in growing follicle in both sheep and humans and not in the early primordial follicles [81, 82]. In contrast, other groups reported FSHR on OSE [83, 84] and on oocytes [57, 85] and also on carcinoma cells [63, 86, 87]. Both Oktay [82] and Tisdall [81] missed out on FSHR expression in primordial follicles since they used primers spanning exons 8–10 for RT-PCR and in situ hybridization which are specific to G-protein coupled, canonical FSHR1 receptor and are spliced out in alternatively spliced growth factor type 1 FSHR3 receptor [31]. Interestingly Zheng et al. [83] used primer sequences from exons 1–5 (which are common in both FSHR1 and FSHR3) of FSHR gene to demonstrate FSHR in the OSE. Meduri et al. [88] showed FSHR (not LHR) binding to human oocytes of primary follicles at both protein and mRNA level (20 folds more than on granulosa cells). FSH binding to oocytes was shown by autoradiography, oocytes responded by mobilization of Ca2 + and authors suggested a direct action of FSH on oocytes development. They used anti FSHR antibody raised against the extracellular domain of FSHR and a 366 bp FSHR transcript (using primers from exons 7 and 10) was detected in granulosa cells whereas a 191 bp product against FSHR (using primers from exons 2 and 4) was detected in the oocytes. *The concept that early follicle growth is gonadotropin independent may not necessarily be true.*

### Pharmacological high rFSH used in vitro cannot be equated to physiological or pathophysiolo-gical observations. Lack of sequencing data on PCR products [71]

More studies in this direction need to be undertaken. Besides reporting a direct effect of FSH on ovarian stem cells in vitro [17, 18], we have also reported studies on intact mice which when treated with FSH result in increased numbers of stem cells in ovaries [89] and testes [19]. Sequencing data of the PCR products in case of Fshr detection in endometriotic tissue has been published [90].

### Why FSHR reported in multiple extragonadal tumor tissues whereas the corresponding normal tissues do not express FSHR [71]

**Fig. 5:**
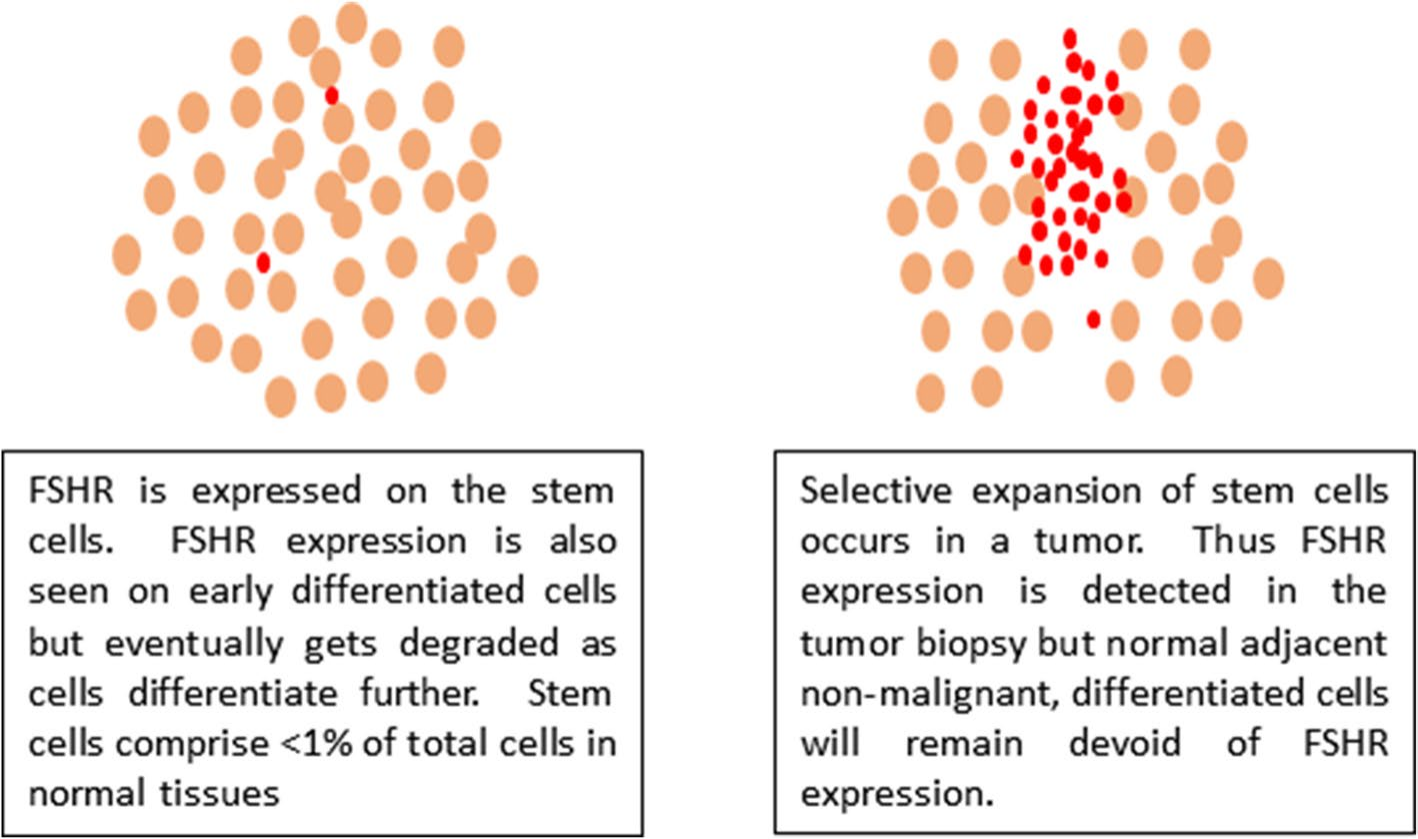
Adjacent mature cells remain negative whereas the cancer cells express FSHR

In order to explain this ambiguity, it becomes pertinent to first understand what is a tumor! Tumor/cancer occurs due to selective, uncontrolled expansion of primitive and tissue-resident stem cells whereas their differentiation is affected. Thus, cancer comprises undifferentiated, primitive cells including stem cells which express abundant FSHR (Fig. 5).

Adult tissues comprise fully differentiated cell types where Fshr transcript may not be detected and even protein may or may not be observed. This is the reason why tumor cells express FSHR and Fshr expression is not detected in the corresponding normal tissue.

As an example, FSHR is known to be expressed on the Sertoli cells in testes and we have reported its expression on testicular stem cells [19]. But FSHR is not observed in the germ cells and sperm that arise by the differentiation of stem cells. This shows that the differentiated cells may not express Fshr/FSHR but it is expressed on stem cells and during early differentiation. Similarly, nuclear OCT-4A is a marker for pluripotent stem cells and is expressed on the stem cells in both ovary and testis. But as soon as the stem cells divide into tissue-specific progenitors, OCT-4 becomes cytoplasmic and eventually gets degraded as the progenitors further differentiate into germ cells.

### Why FSHR are detected in endometrial and endometriotic tissue? Is it real? [71]

Increased expression of FSHR is reported in the secretory phase human endometrium compared to proliferative phase [91]. FSHR expression was reported on human endometrium, fallopian tubes, cervix, myometrium, and placenta [91]. This was shown by immuno-localization studies and was not confirmed by RT-PCR or by Western blotting. Robin et al. [92] reported FSHR expression on endometriotic tissue by immuno-histochemistry studies. Ponikwicka-Tyszko et al. [90] reported functional expression of FSHR in endometriotic lesions. They reported FSHR expression in secretory phase endometrium at both mRNA and protein level in glandular epithelium and stromal cells. Endometriotic lesions in recto-vaginal region and near the ovaries also expressed FSHR and upregulated CYP19A1. Erβ was increased in endometriotic tissue compared to normal endometrium. Upon treatment with recombinant human FSH, endometriotic tissue explants showed increased estradiol production, CYP19Ai and Erβ and FSHR on stromal cells. Sacchi et al. [93] further confirmed functional gonadotropin receptors (FSH, LH/hCG) on human endometrium. FSH and LH/hCG receptors were studied by RT-PCR followed by sequencing and by IHC on glandular epithelium. RT-PCR product was sequenced and cAMP was increased after treatment in primary cultures.

Genesis of above-mentioned results lies in appreciating that stem cells are actively involved in endometrial biology and differentiate to prepare a receptive endometrium, thus stem cells and early epithelial progenitors expressing Fshr/FSHR are more abundant in the secretory phase and thus get easily detected. Similarly, endometriosis is a stem cell disease and epithelial progenitors are increased in numbers which express FSHR. We recently reported maximal numbers of VSELs in the estrus and metestrus stages of estrus cycle [77] and that FSH treatment directly acts on endometrial [76] and myometrial [94] stem cells.

## Salient points

- FSH, secreted by the pituitary is central to mammalian reproduction. As per current understanding, FSH acts directly on the gonads, on the granulosa cells of pre-antral follicles in the ovaries and on Sertoli cells in testes to support follicular growth and spermatogenesis respectively. But evidence is emerging to suggest that FSH acts via alternately spliced isoforms of FSHR to express its pleiotropic effects. FSHR1 is the canonical isoform that acts via cAMP whereas FSHR-3 is growth factor I receptor that acts via MAPK pathway. No biological functions are attributed to FSHR2 and FSHR4 as yet.
- Mutations and SNPs in FSH/FSHR have been studied extensively to investigate their role in various biological processes (OHSS, dose of FSH to stimulate the ovaries in an ART clinic etc.) as well as in pathologies (PCOS, POI, infertility, endometriosis, fibroids, reproductive tissue cancers etc.). But despite decades of research and numerous publications, no significant understanding of clinical relevance has emerged so far. Alternately spliced FSHR isoforms are likely to have a more significant role. But much more research is required in this direction.
- FSHR are expressed on tissue-resident stem/progenitor cells in multiple organs and eventually get degraded as cells differentiate further. Expression of FSHR isoforms is associated with proliferation of stem/ progenitor cells, in normal and cancerous tissues.
- OMICS studies have failed to determine any defect in FSH/FSHR in various pathologies since they involve extracting DNA/RNA/proteins from intact tissues and as a result the contribution of stem cells remains masked as they comprise less than 1% of total cells.
- FSHR are expressed on cancer cells which arise due to excessive and selective self-renewal of tissue-resident stem/progenitor cells whereas adjacent somatic, mature cells evidently remain negative for FSHR. For similar reasons, endometriotic ectopic tissues express FSHR.
- Selection of primers specific to Exon 10 of FSHR (ignoring other alternately spliced isoforms) has resulted in existing (faulty) paradigms regarding FSH/FSHR biology in the ovaries. This has also led to misperceptions in the field since expression of FSHR-3 could not be studied using primers specific for exon 10 (which are spliced in FSHR-3). Early follicle growth is not independent of gonadal action rather Fshr-3 plays crucial role in activating ovarian stem cells to undergo neo-oogenesis to form primordial follicle which also express FSHR (FSHR-3).
- Existing conundrums and known unknowns regarding FSH/FSHR biology can be straightforwardly explained by appreciating FSHR expression on tissue-resident stem cells in multiple adult organs including reproductive tissues.
- To conclude, extragonadal expression of FSHR is not an artefact but basic stem cells biology in adult tissues needs to be appreciated and further investigated.

## Data Availability

It should be present and appropriate for data policy associated with the journal (stated in the submission guidelines). Not applicable.
